# Developing Vaccines for SARS-CoV-2 and Future Epidemics and Pandemics: Applying Lessons from Past Outbreaks

**DOI:** 10.1089/hs.2020.0043

**Published:** 2020-06-17

**Authors:** John Billington, Isabelle Deschamps, Stanley C. Erck, Julie L. Gerberding, Emmanuel Hanon, Sabrina Ivol, John W. Shiver, Julia A. Spencer, Johan Van Hoof

**Affiliations:** John Billington, JD, MPH, is Director, Science Policy, and Emmanuel Hanon, PhD, DVM, is Senior Vice President, Head of R&D; both at GSK Vaccines, Wavre, Belgium.; Isabelle Deschamps, PhD, is Head of Global Vaccine Public Affairs; and John W. Shiver, PhD, is Senior Vice President R&D; both with Sanofi Pasteur, Lyon, France.; Stanley C. Erck, MBA, is President and Chief Executive Officer, Novavax, Gaithersburg, MD.; Julie L. Gerberding, MD, MPH, is Executive Vice President and Chief Patient Officer, Strategic Communications, Global Public Policy, and Population Health; and Julia A. Spencer, PhD, is Associate Vice President, Global Public Policy; both with Merck & Co., Inc., Kenilworth, NJ.; Sabrina Ivol is Senior Specialist, Policy; and Johan Van Hoof, MD, is Managing Director; both with Janssen Vaccines & Prevention BV, Janssen Pharmaceuticals R&D, Leiden, Netherlands.

**Keywords:** Epidemic management/response, Infectious diseases, Vaccine development, SARS-CoV-2, COVID-19

## Abstract

The COVID-19 pandemic is a stark reminder of the heavy toll that emerging infectious diseases (EIDs) with epidemic and pandemic potential can inflict. Vaccine development, scale-up, and commercialization is a long, expensive, and risky enterprise that requires substantial upfront planning and offers no guarantee of success. EIDs are a particularly challenging target for global health preparedness, including for vaccine development. Insufficient attention has been given to challenges, lessons learned, and potential solutions to support and sustain vaccine industry engagement in vaccine development for EIDs. Drawing from lessons from the most recent Ebola epidemic in the Democratic Republic of the Congo, as well as the 2009 H1N1 influenza, 2014-2016 Ebola, and 2015-16 Zika outbreaks preceding it, we offer our perspective on challenges facing EID vaccine development and recommend additional solutions to prioritize in the near term. The 6 recommendations focus on reducing vaccine development timelines and increasing business certainty to reduce risks for companies. The global health security community has an opportunity to build on the current momentum to design a sustainable model for EID vaccines.

The COVID-19 pandemic caused by the SARS-CoV-2 virus is a stark reminder that emerging infectious diseases (EIDs) with epidemic and pandemic potential can inflict heavy human and economic costs and can endanger lives, disrupt societies, and damage economies.^1,2^ The 2014-2016 Ebola epidemic in west Africa served as a wake-up call for the global health security community, showing that even outbreaks in remote villages can have worldwide impact. Indeed, the unpredictable, heterogeneous, and fast-paced nature of some highly transmissible EIDs makes them a particularly challenging target for global health preparedness, including for vaccine development. Unfortunately, today's vaccines cannot be developed “on demand” in response to a surprise threat. Under ideal circumstances, it will take at least 12 to 18 months to bring a safe and efficacious vaccine for SARS-CoV-2 to market. Vaccine development, scale-up, and commercialization is a long, expensive, and risky enterprise that requires substantial upfront planning and offers no guarantee of success.^3^^,^^4^

Nevertheless, there is reason for optimism. The licensure of the world's first Ebola virus vaccine by European, US, and some African regulatory authorities in late 2019, along with prequalification by the World Health Organization (WHO) in record time, marked a groundbreaking milestone for global preparedness.^5^^,^^6^ Regulatory authorities have made extraordinary efforts to help bring safe and effective vaccines to market faster in response to emergencies. The WHO launched a new health emergencies division that includes EID monitoring response, and it drafted a research and development (R&D) blueprint that prioritizes pathogens with epidemic potential.^7^ Gavi, the Vaccine Alliance, adapted its model to include stockpiling of Ebola vaccines.^8^ At the same time, national and regional government programs like the US Biomedical Advanced Research and Development Authority (BARDA)^9^ and the European Union's Innovative Medicines Initiative (IMI)^10^ have sustained their commitment to R&D for EID response. Finally, new entities like the Coalition for Epidemic Preparedness Innovations (CEPI) are galvanizing global pandemic preparedness and response for SARS-CoV-2 and future epidemics and pandemics, using innovative approaches, including investments in novel platform technologies.

The vaccine industry has the experience, capabilities, and capacities required to develop and help deliver critical vaccines to the people who need them under accelerated timelines. A new “golden age” in vaccinology opens doors to greater speed, versatility, and more efficient vaccine platforms as well as more nimble manufacturing capabilities for vaccine development.^11^^,^^12^ Vaccine manufacturers have a demonstrated track record of responding to EIDs when called upon. However, responding to immediate requests to develop vaccines for emergencies often requires manufacturers to put other essential, lifesaving programs on hold. It also carries considerable business, reputational, and liability risks, including both direct and indirect financial impacts.

Much has been written about how we might be better prepared for the next epidemic or pandemic. However, insufficient attention has been given to challenges, lessons learned, and potential solutions to support and sustain vaccine industry engagement in vaccine development for EIDs. Drawing from lessons from the most recent Ebola epidemic in Kivu, Democratic Republic of the Congo (DRC), as well as the 2009 H1N1 influenza, 2014-2016 Ebola, and 2015-16 Zika outbreaks preceding it, we offer our perspective on challenges facing EID vaccine development and recommend additional solutions to prioritize in the near term. A complete solution set must also include considerations for manufacturing, scale-up, vaccine distribution, and access, which are equally if not more challenging for EID response. However, these important aspects of EID preparedness and response are beyond the scope of this article.

## Lessons from Past Outbreak Responses

In this section, we draw on prior experience to show how 2 critical factors work against vaccine development in an emergency: *tim*e and *risk*.

### Time: Vaccines Came Too Late

**Figure 1a.**
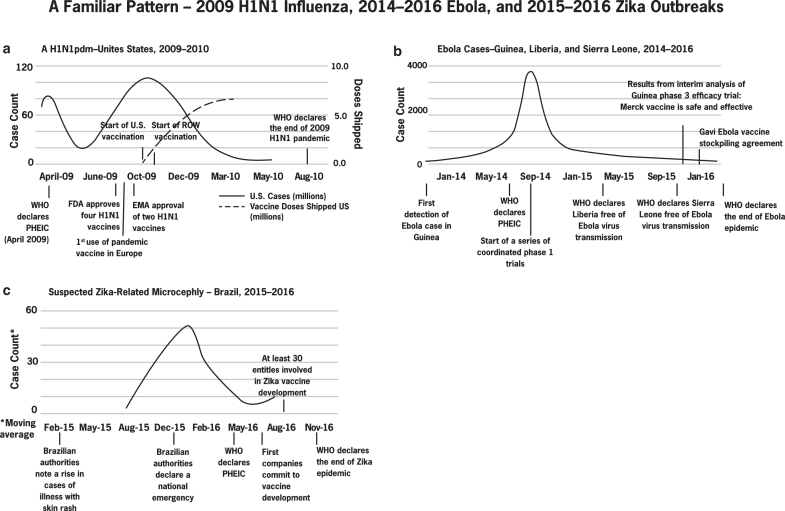
U.S. Centers for Disease Control and Prevention. Update: Novel Influenza A (H1N1) Virus Infections — Worldwide, May 6, 2009.^14^ Figure 1b. U.S. Centers for Disease Control and Prevention. 2014 Ebola Outbreak in West Africa Epidemic Curves.^15^ Figure 1c. Pan American Health Organization and World Health Organization. Zika – Epidemiological Update.^16^

In the event of a widespread epidemic or pandemic, a delay in the availability of a vaccine can result in substantial human and economic loss.^13^ In past outbreaks, vaccines came too late to have a significant impact on the epidemic curve despite extraordinary measures taken to accelerate the responses (Figure 1).

When the 2009 H1N1 influenza pandemic emerged, the vaccine industry had a head start because the seasonal influenza vaccine business and the requisite manufacturing infrastructure were well established. Notwithstanding these advantages, and despite strong multisectoral collaboration and communication involving 6 companies, vaccines were not available in time (the delay was in part due to the wait for the BSL-2 reassorted virus, import permits, and a 5-month production cycle).^17^ Although 4 vaccines based on the new strain were approved by the US Food and Drug Administration (FDA) and shipped globally just 4 months after WHO had declared H1N1 a public health emergency of international concern (PHEIC), they came too late and in insufficient quantities to prevent the estimated 151,700 to 575,400 deaths that occurred worldwide in the first year of the pandemic.^18^

While seasonal influenza has a predictable and sufficient annual market volume to motivate sustained company involvement, it has been nearly impossible to predict the need or demand for EID vaccines. In 2014, when the Ebola epidemic erupted from a remote village in West Africa, there were no approved or late-stage Ebola vaccines available to use. Ebola was a familiar pathogen, but the incidence and geographic spread of this epidemic was unprecedented, demonstrating that even the most isolated outbreaks cannot be ignored.

Within months, the epidemic had spread from Guinea to neighboring Liberia, Nigeria, and Sierra Leone, marking the largest Ebola outbreak to date. When WHO declared a PHEIC in August 2014, several companies acted to expedite the development of early-stage vaccine candidates that were financed in part by public research funding, especially from Canada, the United States, and the European Union.^10^ Companies advanced vaccine candidates through coordinated phase 1 safety trials starting in September 2014 over several sites across Africa, Europe, and North America at unprecedented speed with help from WHO and other partners. But the epidemic began to wane before most could complete phase 2 efficacy trials. One company completed positive interim analyses of phase 3 efficacy trials in Guinea just prior to the end of the PHEIC in December 2015.^19^ By this time, more than 28,500 people were infected and 11,300 had died.^20^

Like the Ebola virus, the Zika virus was discovered decades ago but was thought to cause only sporadic, mild, and self-limited symptoms in Africa and Asia. A vaccine was not considered a public health priority. In the well-documented outbreak in 2015, it was the rapid rise of microcephaly in babies born to Zika-infected mothers in Brazil that brought this disease to global prominence. As the virus spread, fear of this previously unknown complication of infection motivated WHO to declare a PHEIC in February 2016.

Again, vaccine researchers and developers responded to the call, leveraging what scientists knew about Zika from work they were already doing with dengue virus, another flavivirus transmitted by the same species of mosquito that transmits Zika virus. One company partnered with the US government to advance the first Zika virus vaccine candidate to a phase 2 clinical trial in just 5 months.^21^ Shortly thereafter, a second company began the development of its candidate with support from BARDA, and other companies started development programs at their own expense. In a story that repeats itself, however, vaccines could not be developed in time to reduce the disease burden before WHO ended the PHEIC in November of that year. The disease became endemic—acute disease declined as populations were inoculated by the disease. By then, more than 2,000 babies, most of them in Brazil, had been diagnosed with irreversible microcephaly or other neurological disorders.^22^ Although several Zika virus vaccine programs continue today, the low transmission rates and high population-level immunity pose significant obstacles for clinical development.^23^

### Risk: Companies Incur Risks Without Return

Vaccine development involves a substantial investment and a high risk of failure. Typical vaccine development programs from discovery to licensure can cost companies upwards of a billion dollars, can take over a decade to complete, and on average have a 94% chance of failure.^24,25^ While responding to global infectious disease emergencies is central to the vaccine industry's public health mission, business leaders may not always act, and shareholders may not always approve the necessary investments, because the business risks of EID vaccine development tend to outweigh any return on investment. Companies have capacities but tend to be sized for the capacity they need to support their in-line portfolio and their pipeline, running at capacity across the value chain. As such, companies that respond to an outbreak have had to divert resources from core business lines, often for many years, to develop a vaccine that tends to lack a commercial market and may only be needed in limited supply and during limited periods of time. This diversion can cripple all but the largest and most well-supported efforts and creates a major disincentive.

The Ebola and Zika experiences illustrate 3 problems with how governments, philanthropic donors, and nongovernment organizations (NGOs) currently contribute funding for EID vaccine development. First, there are only limited public financial resources to support development in advance of outbreak. The US National Institutes of Health (NIH) funds research on diseases that pose a potential US threat, such as Zika virus, but funding dedicated to accelerating product development tends to be made available only after WHO has declared a PHEIC. Similarly, other government-led initiatives like BARDA in the United States and the IMI in Europe played a substantial funding role in response to recent EIDs like Ebola virus and Zika virus, but this funding is cyclical (eg, dependent on US annual appropriations) and subject to political influence and competing priorities. The creation of CEPI, as discussed further below, is a promising step forward in this regard.

Second, the funding tends to cover only a fraction of the direct vaccine development costs and little to none of the opportunity costs. Importantly, opportunity costs that result from delayed commercially viable programs, diverted manufacturing lines, and diversion of scientists, regulatory team members, and other resources can quickly outweigh direct costs.^26^ Finally, funding tends to be short-term or redirected after the emergency is over. These decisions are made without consideration for the ongoing costs companies will incur in completing development, securing licensure, fulfilling post-market regulatory obligations, and scaling manufacturing appropriately to meet uncertain demand. This short-term funding may also favor first-comer candidates over second- and third-line innovations that may ultimately provide greater value to those at risk.

Companies also face legal and reputational risks when responding to emergencies. For example, during all 3 outbreaks described above, there were concerns about liability risks during clinical stages and emergency use.^21^ Manufacturers are often asked to make the vaccine candidates available on preliminary safety data based on the principle that the benefits of expediting clinical development typically outweigh the risks. However, this principle is not uniformly reflected in regulatory policy and public opinion.

Finally, vaccine companies face a high risk of failure and potential reputational harm, especially when expediting development programs in an emergency environment and under intense public scrutiny. Even with earlier identification of pathogens and accelerated development timelines, vaccine development with the newest technologies and under the best circumstances will take at least 12 to 18 months, and there is a need to appropriately inform public expectations. Epidemic fluctuations also may make it impossible to demonstrate vaccine efficacy before approval and implementation. Despite this uncertainty, companies must continue development without a clear regulatory pathway. These factors negatively affect company risk/benefit assessments for engagement and increase uncertainty for managers and investors, potentially discouraging companies from responding to the next EID.

## Recommendations to Improve the Vaccine Development Ecosystem

To ensure we are prepared to meet the challenge of EIDs, the world needs sustainable solutions that consider the substantial complexity, public health burden, and business risks posed by these unpredictable pathogens. Experience with H1N1, Ebola, and Zika exposed critical flaws in the vaccine development ecosystem for EIDs but also triggered new initiatives that may provide a more coordinated global response framework for the future. Here, we offer 6 recommendations for the near term that will improve the global preparedness ecosystem in advance of the next pandemic (Table 1).

**Table 1.** Actions Needed in Near-Term and Progress So Far

	Objective	Progress Since 2014-15 Ebola (not exhaustive)
*Time*: reduce vaccine development timelines	1. Define and streamline the EID/pandemic regulatory pathway.	• WHO Solidarity Vaccine Trial for SARS-CoV-2 • Parallel dossier review for Ebola virus vaccine
	2. Build partnership models that enable more coordination and collaboration end-to-end.	• CEPI partnership model • Event 201 simulation tabletop exercise
	3. Invest in rapid-response platforms and continue to evaluate and fund next-generation approaches.	• BMGF investments in innovative manufacturing platforms • CEPI investments in platform technology
*Risk*: increase business certainty to reduce risks for companies	4. Ensure proactive, predictable, and sustainable vaccine development and lifecycle funding.	• WHO R&D blueprint; target product profile for SARS-CoV-2 vaccines • CEPI prioritization and proactive funding of early phase (up to 2b)
	5. Improve forecasting for demand and manufacturing requirements.	• Gavi advanced purchase commitment for Ebola vaccine
	6. Create a global indemnification model.	• WHO insurance-based indemnification model for investigational products

*Note:* EID = Emerging Infectious Diseases; CEPI = Coalition for Epidemic Preparedness Innovations; BMGF = Bill & Melinda Gates Foundation; WHO = World Health Organization; Gavi = Gavi, the Vaccine Alliance.

### Reduce Vaccine Development Timelines

Despite good faith efforts by industry, governments, and NGOs to expedite vaccine development during prior outbreaks, a recurring lesson was that “faster-than-ever” is still not fast enough. The experience with recent EIDs suggests 3 opportunities to speed things up: reduce regulatory timelines and complexity, improve company and partner coordination, and accelerate manufacturing.

• *Define and streamline the EID/pandemic regulatory pathway.* Despite considerable progress, there is still only 1 licensed Ebola virus vaccine and no licensed Zika virus vaccines. After the Ebola virus disease PHEIC in 2014, both stringent regulatory authorities and local regulators expedited review and approval of the clinical trial protocols for candidate vaccines.^27^ However, they did not address other structural gaps that slowed progress, including the lack of an explicit pathway for local regulators to leverage recommendations from stringent regulatory authorities like the FDA. Such a pathway would enable local regulators to benefit from the stringent regulatory authority expertise while reducing review and approval times at the country level. Ideally, having pandemic vaccines licensed and inspected for quality by key regional stringent regulatory authorities would help facilitate the process. In addition, other methods to infer likelihood of field efficacy (eg, animal models, observational studies) need to be developed, included in guidelines, and accepted with stakeholder support.^28^

In addition, the virus outbreak underscored the importance of pre-aligned protocols and data requirements for phase 3 trials to accelerate clinical studies. In a PHEIC setting, conducting clinical trials is challenging for 2 reasons: (1) nontraditional (ie, not placebo-controlled) trial designs may be favored by local authorities, despite ethical and regulatory controversies; and (2) access to participants and clinical sites is limited. For the Ebola virus outbreak, some regulators from nonaffected countries required placebo-controlled design, whereas regulators in affected countries prohibited this on ethical grounds. Over time, WHO and other key stakeholders developed a protocol with the manufacturer of the leading vaccine candidate, which could be tested without a placebo.^29,30^ In an encouraging development, the WHO Solidarity Vaccine Trial protocol for SARS-CoV-2 vaccines would create a multi-site, randomized, adaptive trial, designed to accelerate the evaluation period.^31^ This important innovation could help speed up initiation of trials, enable comparisons between multiple sites and products, streamline data collection and processing, establish surrogate endpoints, and improve integration for regulatory submissions.^32^

Finally, consideration is needed to ensure that national-level access and benefit-sharing laws do not delay timely access to virus strains and other genetic resources needed for rapid testing and vaccine development.^33^ The critical need for timely data sharing was demonstrated most recently in China with the SARS-CoV-2 strain.^34^

• *Build partnership models that enable more cooperation and collaboration, end-to-end.* Existing partnership models such as the TB drug accelerator and the Collaboration for AIDS Vaccines Discovery (CAVD) demonstrate the potential value of establishing legally secure constructs that foster company collaboration, data-sharing, and sharing of know-how to tackle public health product development challenges.^35^ Mechanisms through which companies can align with each other and with other partners on actionable information-sharing plans outside of outbreak and epidemic periods might help reduce redundancy and improve efficiency, thus enabling manufacturers to leverage all existing data.^36^ This collaborative approach carries both potential benefits for EID preparedness and some potential risks to the companies' core businesses. The approach also may require differentiated application of competition law, tailored specifically to the EID context. To encourage company participation, all partners involved must agree to, respect, and value the intellectual property that individual companies bring to the partnerships. “Germ” games and other simulations like the Event 201 tabletop exercise are one promising way to continue this dialogue and find collaborative solutions.^37^

• *Invest in rapid-response platforms and continue to evaluate and fund next-generation approaches.* Retooling existing manufacturing facilities or building new capacity to respond to EID demand for vaccines is a significant driver of cost and time. The Bill and Melinda Gates Foundation and other public and private organizations are investing through CEPI and PATH and directly in novel manufacturing technologies and platforms that support rapid change over relatively small batches at comparatively low cost without disruption to other vaccines. As demonstrated by current efforts to rapidly develop SARS-CoV-2 vaccines, EID presents a potentially ideal application for these technologies, most of which are still at early stages.^38^ Vaccine technology platforms that prove successful as EID rapid-response platforms could also be used for more commercially viable disease targets.^39^ Efforts to define regulatory expectations and pathways will be needed, in addition to buy-in from regulators. In the meantime, shared financial commitments from industry, governments, and NGOs are needed to maximize the utility of existing production and stockpiling capacity.

### Increase Certainty to Reduce Business Risks

Epidemic and pandemic preparedness is a high-risk business with uncertain returns. Steps to reduce perceived and actual risk and ensure sustainability could increase company engagement.

• *Ensure proactive, predictable, and sustainable vaccine development and lifecycle funding.* The business case for EID vaccine development can be improved by changing the way government and other funders finance these programs. Funding must be proactive, early, and based on clear criteria for prioritization. The WHO research and development blueprint for EID is an important step forward for pathogen prioritization: Prior work on SARS and MERS coronavirus vaccines (both listed in the blueprint) has proven beneficial in the race for a SARS-CoV-2 vaccine. However, the existence of many different (and long) lists illustrates the difficulty of defining and anticipating which pathogens need a vaccine first and for which population.^40,41^

The launch of CEPI in 2017 marked a watershed moment in the history of global pandemic preparedness. For the first time, there is a multisectoral, global organization dedicated to funding and managing EID early-phase vaccine development for prioritized pathogens drawn from the WHO blueprint. CEPI has secured substantial investments from a global coalition of donors and is accessing innovative financing mechanisms to accelerate grant making.^42^ Industry partners played an active role in the conceptualization and design of CEPI, fully supportive of the vision for a stronger, more nimble, well-resourced public-private partnership. This vision is now materializing as CEPI responds to SARS-CoV-2 with several vaccine programs in development.^43^ Although CEPI is currently resourced only to advance candidates to phase 2b, it has the ambition and potential to eventually support end-to-end solutions, including manufacturing and scale-up. To fully realize this goal, however, it is important that the vaccine industry retain an equal seat at the table with other global stakeholders to set strategy and deliver on these promises.

• *Improve forecasting for demand and manufacturing needs.* Supply and demand forecasting are challenges for routinely used vaccines and are far more complex for EID vaccines because of the highly unpredictable demand, across unpredictable markets, over time. Lessons from the 2009 H1N1 influenza pandemic and Ebola virus outbreaks highlight the importance of establishing priority pathogen lists and terms for business transactions between industry and vaccine purchasers *before* there is an emergency. For vaccine manufacturers, it is difficult to anticipate potential demand and align those forecasts with manufacturing plans. For H1N1 influenza, this risk was compounded by hastily negotiated supply contracts, many of which went unfulfilled when the early waves of the pandemic waned.^44^ Manufacturing capacity is planned during the development process, since the facilities—not just the vaccine itself—are a component of the licensure. Any additional capacity that is needed must be built and approved by all countries receiving vaccine before it can be used.

In 2016, Gavi, the Vaccine Alliance, announced an advanced purchase agreement to help secure a stockpile of an Ebola virus vaccine candidate that had not yet been licensed. Even though this procurement underestimated the volume needed for the DRC epidemic, lessons learned from this construct could inform how large-scale, permanent solutions for other future EID vaccines might be designed. In response to SARS-CoV-2, Gavi is exploring the use of its innovative financing mechanisms to incentivize R&D and reduce manufacturing risk.^45^

• *Create a global indemnification mechanism.* Liability exposure is a serious risk that can deter investment in pandemic vaccines. Without protection from some types of broad liability claims, companies may not be willing to make these vaccines available without complete development data, as these claims could lead to a substantial confidence loss, business loss, and even bankruptcy. Global legal mechanisms are needed to indemnify companies during emergencies when regulatory requirements are followed. WHO, through consultations facilitated by the World Economic Forum with CEPI and Harvard Global Health Institute, recently developed an insurance-based indemnification model to facilitate emergency deployment of experimental vaccines.^46^ This is a promising step forward and should be expanded beyond the clinic, in collaboration with industry and other stakeholders.

## Conclusion

As inventors and producers of vaccines that prevent diseases, vaccine companies are essential partners in all efforts to prepare for and better respond to epidemics, pandemics, and emerging infectious diseases. This work is expensive, risky, and time and labor intensive, and it incurs substantial opportunity costs. A deeper understanding of the ongoing technical, policy, manufacturing, and field-level challenges is needed to inform policy, funding, and program decisions. Critically, the solution must include support for manufacturing scale-up, new regulatory models, and strong end-to-end collaboration with all partners sharing risks and benefits. The licensure and roll-out of the first Ebola virus vaccine is a testament to progress made over the past decade. While the trajectory of SARS-CoV-2 remains uncertain, the mobilization of governments, industry, and funders to develop vaccines and therapies demonstrates a common, global commitment to respond to pandemics. The global community has an opportunity to build on this momentum to design a sustainable model for EID vaccines.
